# Enhancement of keratinocyte growth factor potential in inducing adipose‐derived stem cells differentiation into keratinocytes by collagen‐targeting

**DOI:** 10.1111/jcmm.17619

**Published:** 2022-11-21

**Authors:** Zahra Amidzadeh, Setayesh Yasami‐Khiabani, Hamzeh Rahimi, Shahin Bonakdar, Davoud Shams, Mahdi Habibi‐Anbouhi, Majid Golkar, Mohammad Ali Shokrgozar

**Affiliations:** ^1^ National Cell Bank of Iran Pasteur Institute of Iran Tehran Iran; ^2^ Department of Parasitology Pasteur Institute of Iran Tehran Iran; ^3^ Department of Molecular Medicine, Biotechnology Research Center Pasteur Institute of Iran Tehran Iran

**Keywords:** adipose‐derived stem cells, collagen‐binding domain, differentiation, keratinocyte growth factor, keratinocytes

## Abstract

Different growth factors can regulate stem cell differentiation. We used keratinocyte growth factor (KGF) to direct adipose‐derived stem cells (ASCs) differentiation into keratinocytes. To enhance KGF bioavailability, we targeted KGF for collagen by fusing it to collagen‐binding domain from *Vibrio mimicus* metalloprotease (*vibrio*CBD‐KGF). KGF and *vibrio*CBD‐KGF were expressed in *Escherichia coli* and purified to homogeneity. Both proteins displayed comparable activities in stimulating proliferation of HEK‐293 and MCF‐7 cells. *vibrio*CBD‐KGF demonstrated enhanced collagen‐binding affinity in immunofluorescence and ELISA. KGF and *vibrio*CBD‐KGF at different concentrations (2, 10, and 20 ng/ml) were applied for 21 days on ASCs cultured on collagen‐coated plates. Keratinocyte differentiation was assessed based on morphological changes, the expression of keratinocyte markers (Keratin‐10 and Involucrin), and stem cell markers (Collagen‐I and Vimentin) by real‐time PCR or immunofluorescence. Our results indicated that the expression of keratinocyte markers was substantially increased at all concentrations of *vibrio*CBD‐KGF, while it was observed for KGF only at 20 ng/ml. Immunofluorescence staining approved this finding. Moreover, down‐regulation of Collagen‐I, an indicator of differentiation commitment, was more significant in samples treated with *vibrio*CBD‐KGF. The present study showed that *vibrio*CBD‐KGF is more potent in inducing the ASCs differentiation into keratinocytes compared to KGF. Our results have important implications for effective skin regeneration using collagen‐based biomaterials.

## 1 INTRODUCTION

Skin tissue engineering is becoming an increasingly important area in applied regenerative medicine to treat acute and chronic wounds.^1^, ^2^ Stem cells with the potential of self‐renewal and differentiation into multiple cell types have been proposed as promising cell sources for accelerating wound healing.^3^, ^4^ Adipose‐derived stem cells (ASCs) are of mesenchymal origin with extreme differentiation plasticity and immunosuppressive effect that have been widely used for regenerative medicine.^5^, ^6^

As stem cells can differentiate into many cell types, directing stem cell commitment into the desired lineage is vital for using these cells in clinical applications.^7^ A variety of approaches have been developed to control stem cell differentiation. Manipulating physical factors of scaffolds or using nanoparticles in the culture systems reportedly affected the fate of stem cells.^8^, ^9^ Novel strategies, including genetic engineering of stem cells and epigenetic modification through demethylation agents, also regulate stem cells' differentiation towards specific lineages.^10^, ^11^ Moreover, applying various growth factors (GFs) as potent exogenous therapeutic agents is the conventional method leading to highly specific modulating of lineage commitment of stem cells.^12^, ^13^

Keratinocyte growth factor (KGF) is a crucial mediator in wound healing, which is upregulated dramatically after skin injuries. KGF, also known as FGF7, is a member of the FGF family that acts specifically on epithelial cells by binding to its specific tyrosine kinase receptor (FGFR2‐IIIb/KGFR). This polypeptide stimulates epithelial cell proliferation and is involved in the migration and differentiation of keratinocytes at the initiation stages.^14^, ^15^, ^16^ The activity of KGF as the potent mitogen or differentiation inducer is exerted through its binding to KGFR and prompting subsequent signalling cascades, including Ras‐mitogen‐activated protein kinase (Ras/MAPK) and phosphoinositide 3‐kinase‐Akt (PI3K/AKT) pathways. On the other hand, KGFR activation by high doses of KGF results in negative feedback between the two signalling pathways, which counterbalance proliferation and promote differentiation.^17^

Despite the pivotal role of KGF in wound healing, its application has been restricted due to its short half‐life, low target specificity, and rapid diffusion at the administration site.^18^

Immobilization of GFs on biomaterials is a successful strategy for improving the efficiency and stability of GFs.^19^, ^20^ Administration of GFs in an immobilized form leads to localized delivery and prolonged activity that would lower the required dose and the adverse effects of GFs. Moreover, direct contact of immobilized GFs with cells can enhance bioavailability and cell signalling.^21^

In order to immobilize GFs, protein engineering techniques are used to bind GFs covalently or non‐covalently, through affinity binding, to diverse biomaterials.^20^ Collagen is the main extracellular matrix component commonly used in many skin tissue engineering scaffolds and is gaining much attention as the appropriate target site for the immobilization of GFs.^22^, ^23^

The present study aimed to improve KGF retention in culture conditions and increase its bioavailability by incorporating a collagen‐binding domain (CBD) into the molecule. Although numerous studies produced various recombinant fusion proteins containing different collagen‐binding domains and GFs, few studies applied a CBD‐growth factor for stem cell differentiation.^24^ For the first time, we assessed the effect of a fusion form of KGF having collagen‐binding ability on inducing adipose‐derived stem cells (ASCs) differentiation into keratinocyte lineage. *vibrio*CBD‐KGF, containing KGF at the C‐terminal of the collagen‐binding domain derived from *Vibrio mimicus* metalloprotease,^25^ was produced in *Escherichia coli*. Biological activity and collagen‐binding ability of the fusion protein and KGF were assessed. We used ASCs grown on collagen‐coated culture plates and treated them with *vibrio*CBD‐KGF or KGF. Differentiation of ASCs into keratinocytes was investigated by real‐time PCR analysis and immunofluorescence staining of keratinocytes differentiation markers. Morphological changes and expression levels of stem cell markers in the treated ASCs were also evaluated. Our findings indicated that *vibrio*CBD‐KGF has high collagen affinity and enhanced potency in inducing ASCs differentiation into keratinocytes.

## 2 MATERIAL AND METHODS

### 2.1 Construction of plasmids expressing KGF and *vibrio*CBD‐KGF

DNA sequence of full‐length Human KGF (FGF‐7), encoding a 194‐amino acid polypeptide, was obtained from NCBI Database (GenBank accession no. KF840563). The 31 amino acids of the signal sequence and the first 23 amino acids of KGF, downstream of the signal sequence, were excluded from both constructs of KGF and *vibrio*CBD‐KGF to improve the stability of KGF.^26^ The fusion protein contained KGF and CBD derived from *Vibrio mimicus* metalloprotease, in which CBD was inserted at the N‐terminal of KGF. The two constructs were synthesized by Biomatik (Canada) and sub‐cloned between the restriction sites, *NdeI* and *HindIII*, of pET26b(+) expression plasmid (Novagen, USA).

### 2.2 Expression of KGF and *vibrio*CBD‐KGF

*Escherichia coli BL21* (*pLys* *S*) and *Rosetta‐gami* (*DE3*) strains (Novagen, USA) were used as the expression hosts to produce KGF and *vibrio*CBD‐KGF, respectively. A single colony of transformed bacteria was cultured overnight in 5 ml of Luria‐Bertani broth (LB) medium pre‐mixed with Kanamycin (100 μg/ml) as the primary culture. The culture was then scaled up by inoculating it into 1000 ml of fresh LB medium and incubated until it reached an OD_600_ of 0.6–0.8. The expression of target proteins was induced by adding 0.5 mM IPTG, followed by incubation overnight at 25°C and 30°C for KGF and *vibrio*CBD‐KGF, respectively. Bacteria were harvested from culture media by centrifugation at 6000 × g for 10 min.

### 2.3 Purification of KGF

The bacterial pellet (1.5 g) was re‐suspended in 30 ml of the binding buffer (50 mM phosphate buffer, 50 mM NaCl, pH 7.4) containing 5 mM EDTA and 0.5 mM PMSF. Bacteria were lysed by sonication on ice for 5 min at 200 W with 20 s pulses and 30 s pauses. The cleared lysate was obtained by high‐speed centrifugation at 12,000 × g for 30 min at 4°C and loaded onto Heparin‐sepharose column (Bio‐Rad, USA), previously equilibrated with the binding buffer. The column was washed with a linear gradient of NaCl concentration, and the fraction containing KGF was eluted from the column at 700 mM NaCl. The eluted protein was buffer‐exchanged using G25 desalting column (GE Healthcare, USA) to reduce NaCl concentration to 50 mM and loaded onto SP cation exchange chromatography (GE Healthcare, USA) equilibrated with the binding buffer (50 mM phosphate buffer, 50 mM NaCl, pH 7.4). Unbound proteins and impurities were washed away from the column by passing through the binding buffer and washing buffer containing 100 mM NaCl. Finally, KGF was eluted from the column using elution buffer (phosphate buffer 50 mM, NaCl 300 mM, pH 7.4). The purified protein was stored in aliquots at −70°C until used.

### 2.4 Purification of *vibrio*CBD‐KGF

Cell pellet disrupted by sonication after re‐suspending in the binding buffer (50 mM phosphate buffer, 250 mM NaCl, pH 7.4) containing 5 mM EDTA and 0.5 mM PMSF. Following centrifugation at high speed, the soluble fraction was subjected to HiTrap Phenyl HP column (GE Healthcare, USA) previously equilibrated with the binding buffer. Elution of bound proteins was performed by a linear gradient of NaCl concentration from 250 mM to zero. The fusion protein was eluted at zero concentration of NaCl and stored in aliquots at −70°C until used.

### 2.5 SDS‐PAGE and Western blot analysis

Protein samples with molecular weight markers were loaded onto the wells of 12% SDS‐PAGE gel and separated by running electrophoresis for 2 h at 150 V. Target proteins were visualized by Coomassie blue staining or Western blotting was performed as follows: Protein bands were transferred to PVDF membrane (BioRad, USA). The membrane was blocked with 2% BSA in PBS‐T (Phosphate‐Buffered Saline containing 0.1% Tween 20) at 4°C overnight. The following day, the membrane was incubated with rabbit anti‐KGF antibody (Sigma, USA), diluted (1:1000) in the blocking buffer, for 2 h at room temperature by gentle agitation. After three washes with PBS‐T, the membrane was incubated with goat anti‐rabbit IgG secondary antibody, conjugated with horseradish peroxidase (HRP), at 1:1000 dilution (TransGen Biotech, China) for an hour at room temperature. Following three washes with PBS‐T, the peroxidase activity was visualized by adding the DAB solution (0.5% 3,3‐diaminobenzidine containing 0.15% H_2_O_2_) to the membrane. The membrane was incubated in the dark until the bands appeared.

### 2.6 Cell proliferation assay

Biological activity of KGF and *vibrio*CBD‐KGF was examined by measuring their effects on the proliferation of human HEK‐293 and MCF‐7 cells using MTT (3‐[4,5‐dimethylthiazole‐2‐yl]‐2,5‐diphenyltetrazolium bromide) assay and compared with commercial KGF (Royan Institute for Biotechnology, Iran). HEK‐293 cells were grown in DMEM‐high glucose (4.5 g/L) with 2% L‐glutamine supplemented with 10% fetal bovine serum (FBS) (Gibco, Grand Island, NY) and 1% Penicillin/Streptomycin (Pen/Strep) (Sigma, USA). MCF‐7 cells were grown in DMEM‐low glucose (1 g/L) with 10% FBS and the same antibiotics. For the experiment, HEK‐293 cells at a density of 2000 and MCF‐7 cells at a density of 3000 cells per well were seeded in the 96‐well cell culture plate. In HEK‐293 cells, after 24 h of incubation at 37°C in a 5% carbon dioxide atmosphere, media was replaced by DMEM High Glucose with 5% FBS containing KGF and *vibrio*CBD‐KGF at concentrations of 0.06 to 50 ng/ml. Cells cultured in media with 5% FBS were regarded as the negative control, while cells cultured in high‐serum media (10% FBS) were used as the positive control. MCF‐7 Cells were serum‐starved for 12 h and treated with KGF and *vibrio*CBD‐KGF at concentrations of 0.2 to 50 ng/ml. MCF‐7 cells that were cultivated in media containing 10% FBS and without FBS were considered as the positive and negative controls, respectively.

HEK‐293 cells were cultured for 5 days, and MCF‐7 cells were cultivated for 3 days. Then, cells were incubated with 1 mg/ml MTT (Bio Basic, Canada) for 4 h. Subsequently, media was discarded, and 100 μl of dimethyl sulphoxide (Merck, Germany) was added per well to solubilize the formazan crystals. Finally, the absorbance values at 570 nm with a reference wavelength of 630 nm were measured by a multi‐well spectrophotometer (Power Wave XS, BioTek, USA).

### 2.7 Collagen‐binding assay by immunofluorescence

A 48‐well culture plate was coated with 300 μg/ml of acid‐soluble type I collagen from the bovine tendon (Nano Zist Arayeh, Iran), as described in previous methods.^27^ The plate was blocked with 1% BSA in PBS‐T (PBS and 0.1% Tween 20) for 2 h at 37°C. In the next step, KGF and *vibrio*CBD‐KGF at 50 ng/ml concentration were added into separate wells and incubated at 37°C for 2 h. After extensive washing with PBS‐T, rabbit anti‐KGF antibody (Sigma, USA) was applied at 1:1000 dilution to the plate as the primary antibody. The plate was incubated overnight at 4°C and washed three times with PBS‐T. FITC‐conjugated anti‐rabbit IgG (1:1000, Abcam, UK) was added to the wells as the secondary antibody. Subsequently, the plate was incubated at 37°C for an hour, followed by three washes with PBS‐T. The interaction of proteins with collagen was visualized in the 48‐well plate under fluorescence microscopy (Labomed, USA). Scanning Electron Microscopy (SEM) and Picro‐sirius red staining were used to confirm that collagen was coated and remained on the surface of wells after washing processes.^[^ ^28^, ^29^ ^]^

### 2.8 Collagen‐binding assay by enzyme‐linked immunosorbent assay (ELISA)

A 96‐well ELISA plate was coated with collagen and blocked, as mentioned above. Serial dilutions of *vibrio*CBD‐KGF and KGF (25, 50, 100, 150, and 200 ng/ml) were applied to the plate, followed by incubation at 37°C for 2 h. Wells were washed extensively with PBS‐T, and after adding rabbit anti‐KGF antibody (1:1000, Sigma, USA), the plate was kept at 4°C overnight. After three times washing with PBS‐T, HRP conjugated‐anti‐rabbit IgG (1:1000, TransGen Biotech, China) was applied to the wells and incubated at 37°C for an hour. Following three times washing with PBS‐T, TMB substrate (BioRad, USA) was added (100 μl/well) to each well of the plate and incubated for 10 min to develop the signals. The reaction was stopped by adding phosphoric acid (1 M), and the optical density (OD) values were read at 450 nm with 630 nm as the reference wavelength by spectrophotometer.

### 2.9 Isolation and culture of ASCs

Adipose‐derived stem cells were isolated by adopting the same procedure used by previous reports with some modifications.^30^ Following informed consent, the adipose tissue of patients undergoing abdominal operation was collected and washed extensively with sterile PBS supplemented by 3% Pen/Strep. Connective tissue and blood vessels were removed from the sample using scalpels and scissors, followed by treatment with 0.02 mg/ml collagenase type I (Gibco, Grand Island, NY) at 37°C for 40–60 min. Subsequently, the stromal vascular fraction (SVF) was separated by centrifugation at 300 × g for 5 min. SVF pellet containing ASCs was re‐suspended in the proliferation medium containing DMEM‐F12 (Gibco, Grand Island, NY) with 10% FBS and 1% Pen/Strep and cultured at 37°C, 5% CO2 for 2 days without changing the media. The presence of surface markers of mesenchymal stem cells (MSCs) in isolated ASCs was investigated by flow cytometry with allophycocyanin (APC) conjugated anti‐CD90, fluorescein isothiocyanate (FITC) conjugated anti‐CD45, phycoerythrin (PE) conjugated anti‐CD105, and anti‐CD34 (BioLegend, USA). Immunophenotyping of cells was performed on BD FACS Calibur flow cytometer (BD Biosciences, San Joes, CA, USA), and data were analysed by FlowJo 7.6.1 software.

### 2.10 ASCs differentiation into keratinocytes

Adipose‐derived stem cells at the second to fourth passages were cultivated and used when they reached about 80% confluency. After trypsinization, the cells were seeded in 12‐well cell culture plates previously coated by collagen type I (300 μg/ml). The cultured cells were maintained in the proliferation medium for 24 h. The media was then replaced with a defined keratinocyte serum‐free medium (KSFM) (Gibco, Grand Island, NY), and the cells were treated with different concentrations of KGF and *vibrio*CBD‐KGF (2, 10, and 20 ng/ml). The media was changed every 72 h during the differentiation process, which lasted 21 days. ASCs grown in the proliferation medium in the absence of KGF or *vibrio*CBD‐KGF were considered untreated control.

### 2.11 Analysis of differentiation markers by real‐time PCR

Total RNA was extracted from the ASCs cells at the end of the differentiation process using Total RNA mini kit (Favorgen, Taiwan). The purity and concentration of total RNA were assessed by NanoDrop spectrophotometer (Thermofisher Scientific, USA). RNA samples were reverse‐transcribed using cDNA synthesis Kit (PCR Biosystems, UK) according to the manufacturer's instruction. Specific primers, shown in Table 1, for amplification of Keratin‐10 (KRT10), Involucrin, collagen type I (Collagen‐I), Vimentin, and GAPDH were used to quantify the expression of differentiation markers by real‐time PCR.^31^ The quantitative PCR reaction was performed using SYBR Green PCR Master Mix (PCR Biosystems, UK) in a Step One instrument (Applied Biosystems, USA). The PCR reaction was carried out by adding 2 μl of cDNA in 10 μl SYBR Green PCR master mix and 200 nM of each primer per reaction. RNase/DNAse‐free water was added to obtain 20 μl final volume. The amplification process was performed in one cycle of 95°C for 2 min, followed by 40 cycles of 95°C for 5 s, 60°C for 25 s, and the final thermal denaturation steps for melting curve analysis. ASCs without treatment were used as negative controls. Relative target gene expression levels in entire samples were calculated based on the 2^−ΔΔCT^ method considering GAPDH as the internal control gene.

**TABLE 1:** Sequence of the primers used in real‐time PCR

Species	Gene	Forward primer	Reverse primer
Human	Involucrin	5′‐TTCCTCCTCCAGTCAATACCCA‐3′	5′‐CTGTGGCTCCTTCTGCTGTTG‐3′
Human	KRT10	5′‐TGAAGAAGAACCACGAGGAG‐3′	5′TGTTCAGCAAGTTGTTCATATTG‐3′
Human	Collagen‐I	5′‐CGATGGCTGCACGAGTCA‐3′	5′‐GGTTCAGTTTGGGTTGCTTGTC‐3′
Human	Vimentin	5′‐TGCTGGAAGGCGAGGAGAG‐3′	5′‐AGGTCATCGTGATGCTGAGAAGT‐3′
Human	GAPDH	5′‐GAGTCCACTGGCGTCTTCA‐3′	5′‐TCTTGAGGCTGTTGTCATACTTC‐3′

### 2.12 Analysis of differentiation markers by immunofluorescence

For immunofluorescence staining, cells were initially fixed with 4% paraformaldehyde (Sigma, USA) for 15 min at room temperature. Fixed cells were permeabilized with 0.2% Triton X‐100 (Sigma, USA) for 10 min and washed three times with PBS. Wells were blocked with 1% BSA (bovine serum albumin) in PBS‐T (PBS containing 0.1% Tween 20) for 30 min to prevent non‐specific binding. The primary antibodies, mouse anti‐KRT10 (Santa Cruz, USA) and rabbit anti‐Involucrin (Biorbyt, UK) diluted in PBS (1:100, Sigma, USA), were applied to the cells and incubated at 4°C overnight. The cells were washed with PBS‐T and incubated with PE‐conjugated goat anti‐mouse and FITC‐conjugated goat anti‐rabbit (Abcam, UK), both diluted at 1:200 for an hour at 37°C. Following washing with PBST, cell nuclei were stained with DAPI (1: 1000, Sigma, USA) for 10 min. The specimens were analysed under a fluorescent microscope (LABOMED, USA), and images were captured and processed using Image J analysis software.

### 2.13 Statistical analysis

Data were expressed as means ± SD and analysed by GraphPad Prism (v.8). One‐way anova and Student's *t*‐test were utilized for evaluation of the statistical significance of differences as appropriate. A *p*‐value <0.05 was considered statically significant.

## 3 RESULTS

### 3.1 Expression and purification of KGF and *vibrio*CBD‐KGF

**FIGURE 1:**
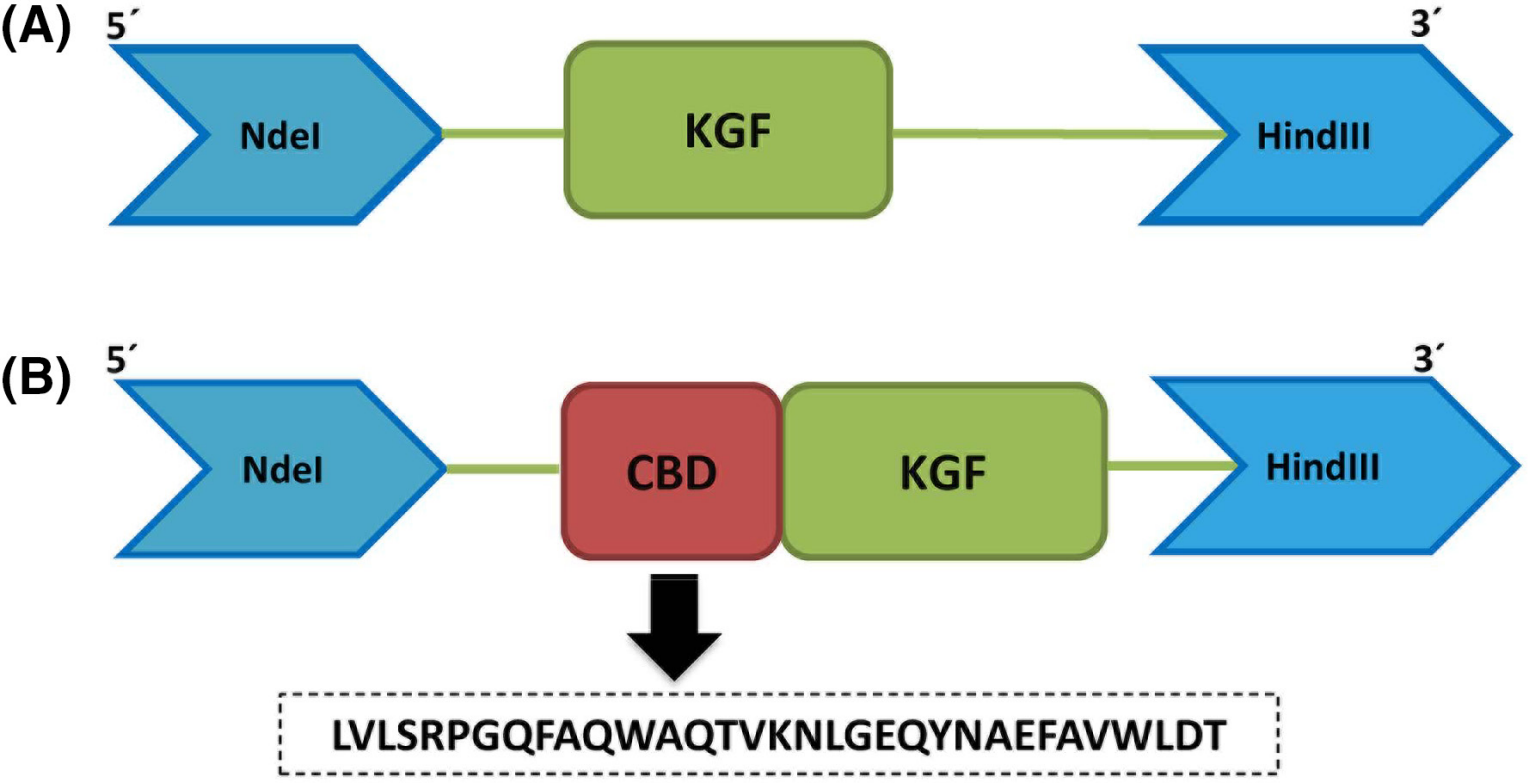
Schematic representation of KGF and *vibrio*CBD‐KGF constructs. (A) KGF construct containing the gene encoding KGF protein with 140‐amino acids. The 31 amino acids of the signal sequence and 23 amino acids, after the signal sequence, at N‐terminal of full‐length KGF (194‐amino acids, GenBank accession no. KF840563) were excluded and it was inserted into the pET26b(+) expression vector. (B) The sequence obtained from the collagen‐binding domain of *Vibrio mimicus* metalloprotease encoding 33‐amino acids was inserted at N‐terminal of KGF (140‐amino acids) and then ligated into a pET26b(+) expression vector.

**FIGURE 2:**
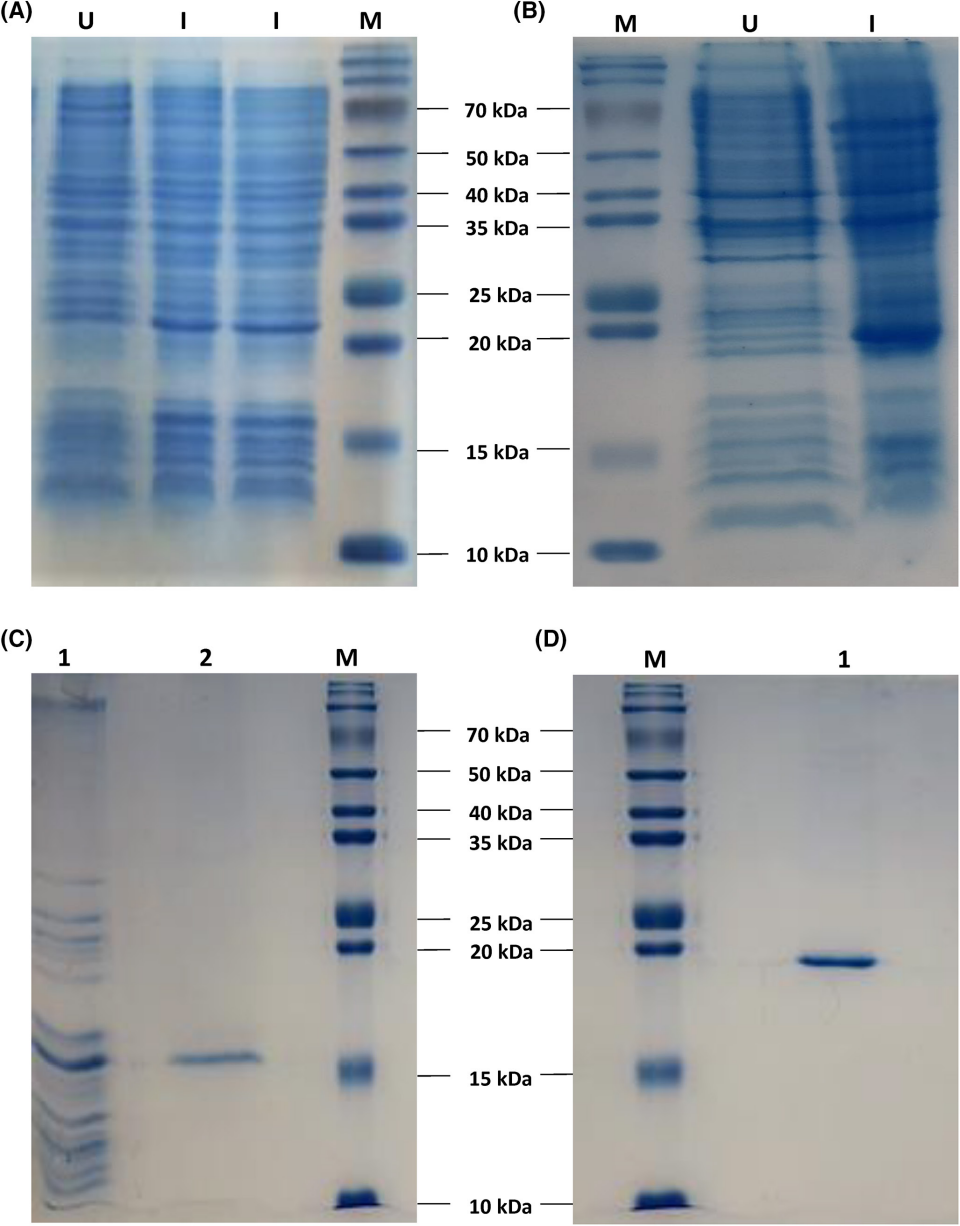
SDS‐PAGE analysis of expression and purification of KGF and *vibrio*CBD‐KGF. Lysates of un‐induced and induced bacteria were electrophoresed under reduced conditions. (A) The expression of KGF in *pLysS* bacteria and (B) *vibrio*CBD‐KGF in *Rosseta gami* bacteria were monitored after Coomassie brilliant blue staining. U, un‐induced; I, Induced, M, protein marker. (C) KGF was purified by heparin‐sepharose affinity chromatography (lane1), followed by SP cation exchange chromatography (lane2). (D) *vibrio*CBD‐KGF was purified by hydrophobic interaction chromatography in a single step (lane1).

**FIGURE 3:**
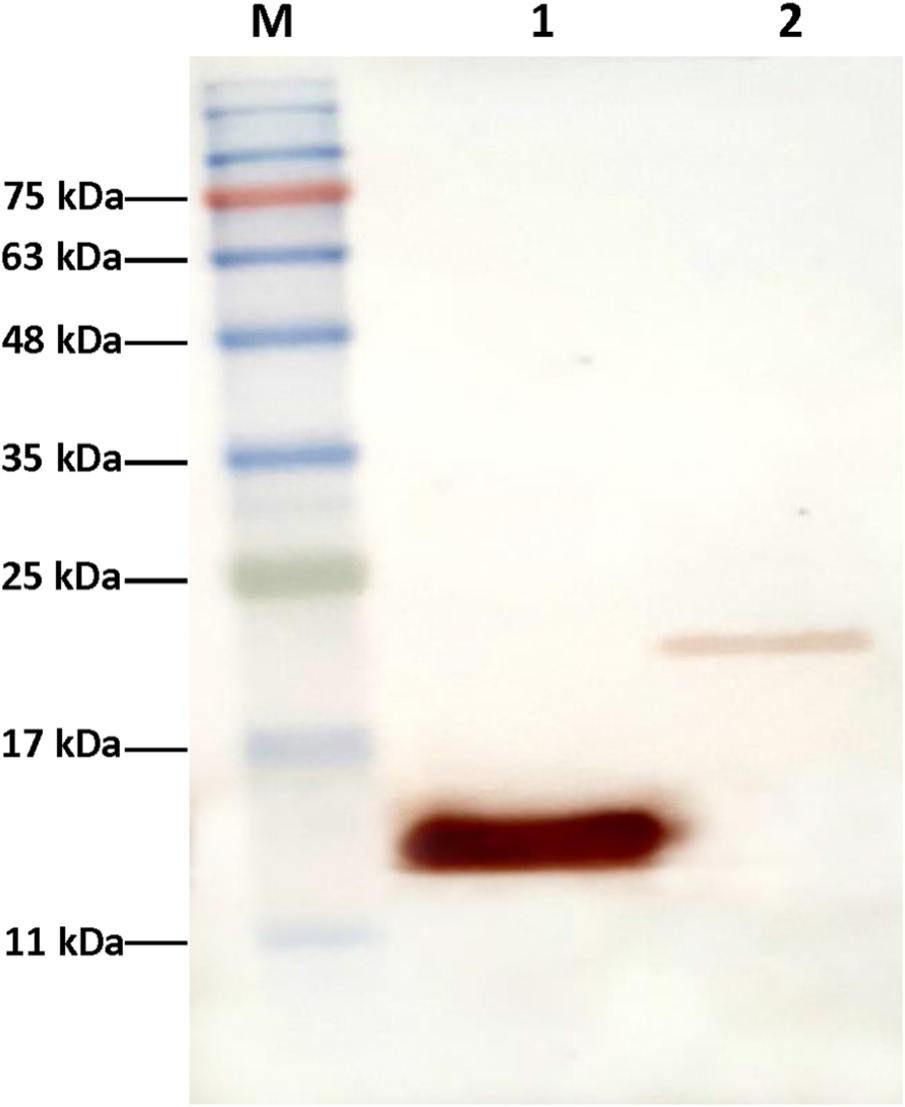
Western blot analysis of purified KGF and *vibrio*CBD‐KGF. Lane M: Protein marker; lane 1: purified KGF; lane 2: purified *vibrio*CBD‐KGF. Western blot analysis was carried out using rabbit anti‐KGF antibody and goat anti‐rabbit HRP conjugated antibody.

The schematic structure of two constructs expressing KGF and *vibrio*CBD‐KGF are shown in Figure 1. In the fusion construct, the collagen‐binding sequence that belongs to *Vibrio mimicus* metalloprotease was inserted at N‐terminal of KGF sequence without any linker. KGF and *vibrio*CBD‐KGF were expressed in *E. coli* (*DE3*) *pLysS* and *Rosetta‐gami* (*DE3*) strains, respectively. Soluble expression of the proteins was increased at temperatures lower than 37°C. After induction with IPTG, cultivation at 25°C for KGF and 30°C for *vibrio*CBD‐KGF resulted in maximum soluble protein expression. SDS‐PAGE analysis showed that the expected bands of KGF (16 kDa) and *vibrio*CBD‐KGF (20 kDa) were present in induced cells (Figure 2A,B). Purification of KGF was performed by Heparin‐sepharose affinity chromatography combined with SP cation exchange chromatography. KGF was partially purified by Heparin‐sepharose affinity chromatography. Further purification of KGF as a single band was carried out by SP cation exchange chromatography (Figure 2C). *vibrio*CBD‐KGF was highly purified by Hydrophobic interaction chromatography in a single step. When soluble lysate passed through the column, most impurities were excluded from the sample in the Flow‐through. Other impurities that bind to the column were removed by decreasing NaCl concentration. *vibrio*CBD‐KGF with high purity eluted in a zero concentration of NaCl (Figure 2D). The identity of the purified proteins was analysed by Western blotting. Although the same concentration of purified KGF and *vibrio*CBD‐KGF were used for Western blotting, the band of *vibrio*CBD‐KGF appeared lately, and its signal was much weaker than KGF (Figure 3). The reason for this is not apparent, but it may be happened due to the inaccessibility of some necessary epitopes for antibody recognition in *vibrio*CBD‐KGF structure.

### 3.2 Biological activity

**FIGURE 4:**
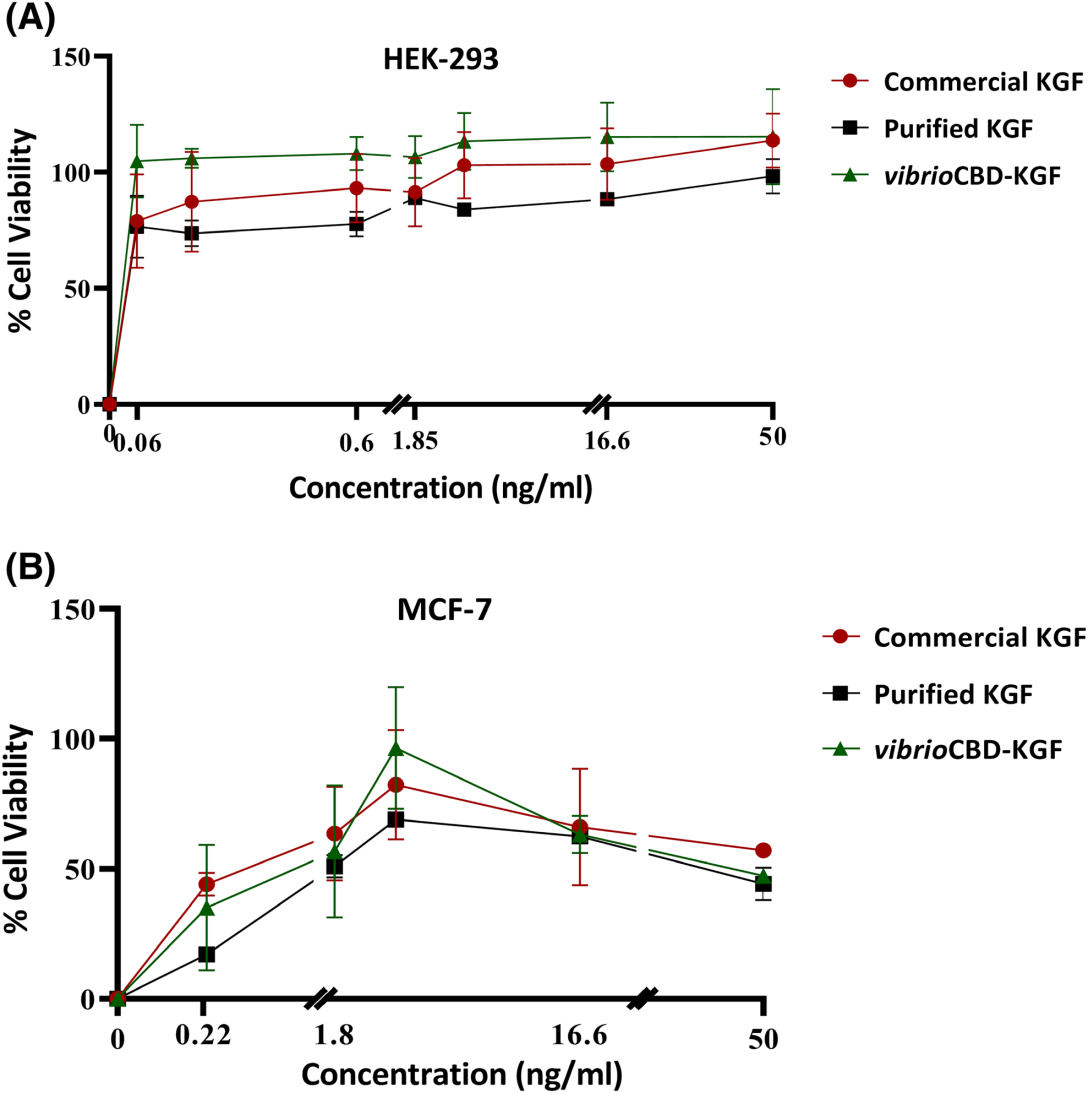
The stimulating effect of purified KGF and *vibrio*CBD‐KGF in HEK‐293 and MCF‐7 cells were assessed and compared with the commercial KGF using MTT assay. (A) HEK‐293 cells were treated with different concentrations of growth factors (0.06–50 ng/ml), and cell proliferation was assessed after 5 days. (B) Mitogenic activity of KGF and *vibrio*CBD‐KGF at concentrations of 0.2–50 ng/ml was also evaluated in MCF‐7 cells. Cells were serum starved for 12 h and, after treatment with different concentrations of proteins, cultivated for 3 days. Both KGF and *vibrio*CBD‐KGF promoted cell proliferation in different concentrations. Data were expressed as means ± SD of proliferation values of triplicate wells.

Cell growth‐promoting activity of KGF and *vibrio*CBD‐KGF was evaluated on HEK‐293 and MCF‐7 cell lines. As shown in Figure 4A, both proteins could stimulate the proliferation of HEK‐293 cells in a dose‐dependent manner. KGF and *vibrio*CBD‐KGF displayed similar results in inducing the proliferation of cells by comparison with commercial KGF. Similarly, MCF‐7 cells showed increases in cell proliferation when treated with different concentrations of KGF and *vibrio*CBD‐KGF (Figure 4B). These findings indicated that CBD moiety did not have adverse effects on the biological activity of KGF.

### 3.3 Collagen‐binding assay

**FIGURE 5:**
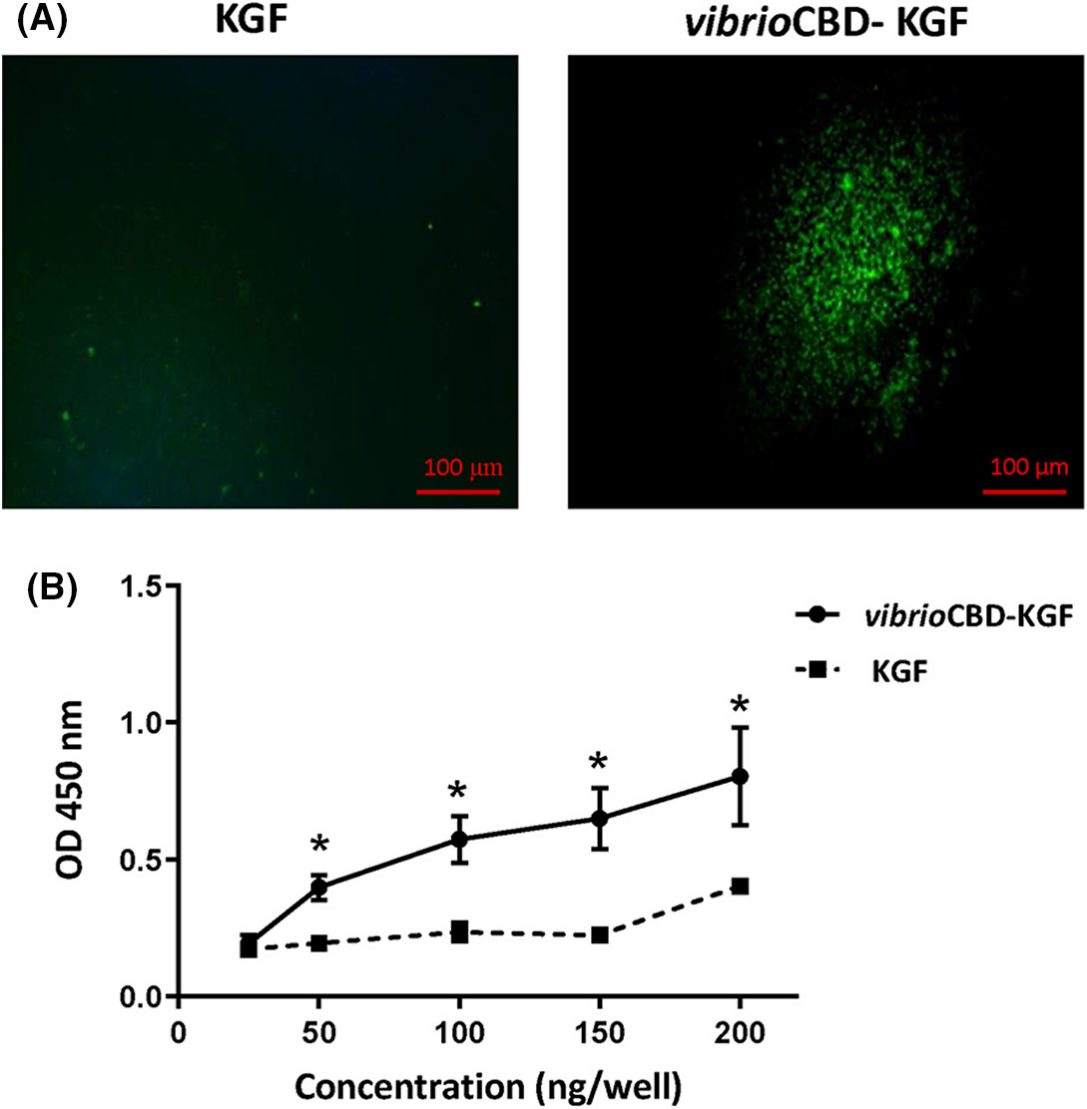
Collagen‐binding assay by immunofluorescence staining and ELISA. (A) Immunofluorescence staining of bound KGF and *vibrio*CBD‐KGF to collagen‐coated wells. The same concentration of KGF and *vibrio*CBD‐KGF were used. Interaction of proteins with collagen appeared as fluorescence spots. (B) Dose‐dependent binding of *vibrio*CBD‐KGF was evaluated by ELISA immunoassay. Different concentrations of *vibrio*CBD‐KGF and KGF (25–200 ng/ml) were added to collagen‐coated wells. The binding levels were measured at OD 450 nm. The assay was performed in triplicate for each concentration. *Statistically different compared to KGF (*p* < 0.05).

The collagen‐binding ability of *vibrio*CBD‐KGF was examined by two different experiments (Figure 5). In immunofluorescence staining, sharp fluorescence spots were visualized only in the well treated with *vibrio*CBD‐KGF, indicating specific interaction of *vibrio*CBD‐KGF and collagen that was coated on the wells. It is also confirmed by SEM analysis that collagen was coated and remains on the surface of wells after washing processes in immunofluorescence assay. The SEM image of a non‐collagen‐coated well was used as the control. Moreover, the bright‐field image of Picro‐sirius red staining proved the presence of collagens, which were seen as red reticular fibres, on the surface of the well (Figure S1).

In collagen‐based ELISA, different concentrations of KGF and *vibrio*CBD‐KGF, from 25 to 200 ng/well, were applied to the ELISA plate coated with collagen, and binding curves were obtained. OD values of wells treated with *vibrio*CBD‐KGF were significantly higher than those treated with KGF at all concentrations. These results showed that *vibrio*CBD‐KGF could interact specifically with collagen that was coated on the wells. The increase in OD value of wells treated with 200 ng/well of KGF might be attributed to the weak interaction of KGF with collagen or non‐specific protein–protein interaction favoured at high protein concentrations.

### 3.4 Isolation and characterization of ASCs

**FIGURE 6:**
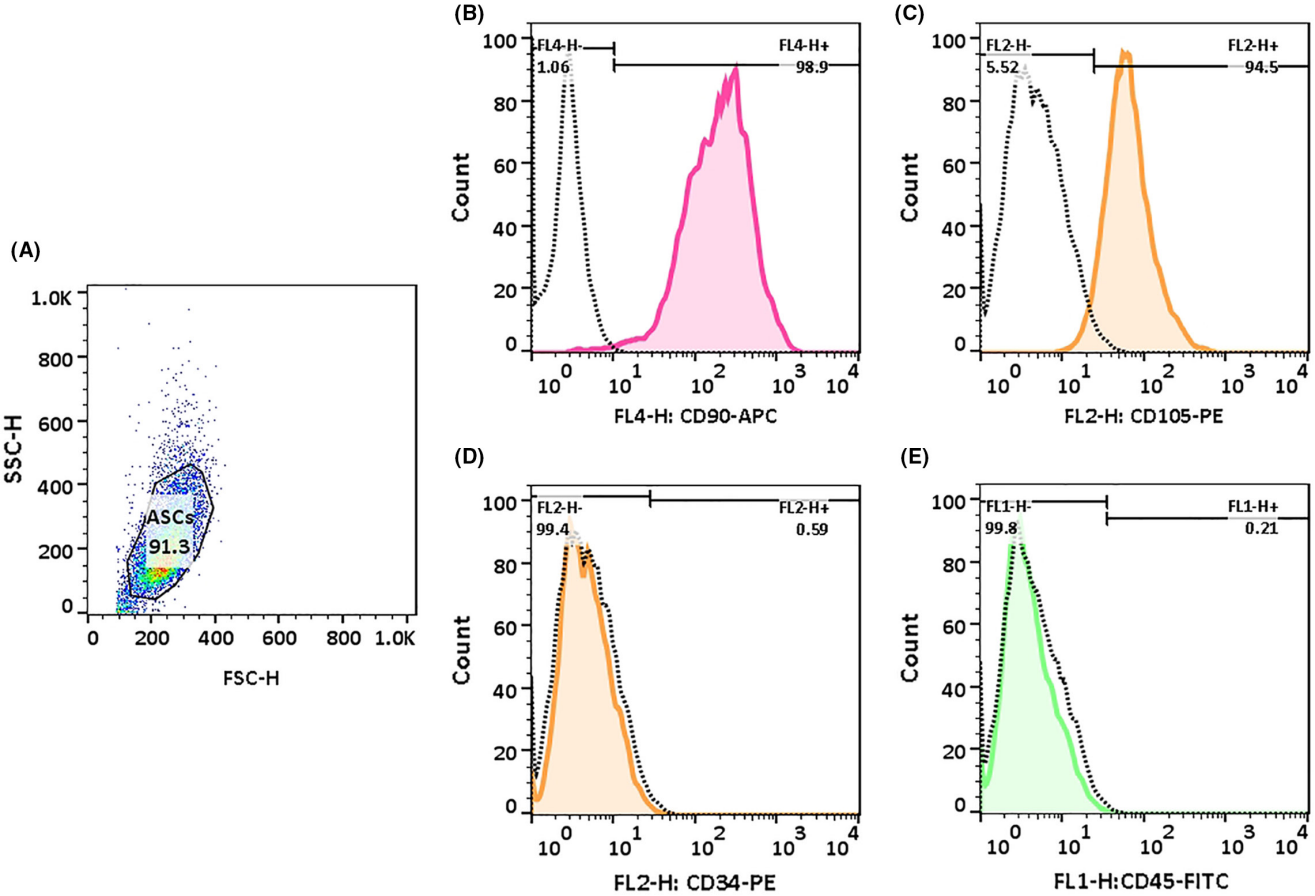
Isolated ASCs at the second passage were characterized by flow cytometry. (A) The FSC/SSC gating graph was used to determine the population of interest. Flow cytometry analysis of cultured ASCs that stained with (B) Allophycocyanin (APC) conjugated anti‐CD90, (C) phycoerythrin (PE) conjugated anti‐CD‐105, (D) anti‐CD34, and (E) fluorescein isothiocyanate (FITC) conjugated anti‐CD45. Dotted lines represent isotype controls. ASCs were positive for mesenchymal stem cell markers (CD90, CD105) and negative for expression of haematopoietic (CD34) and pan‐leukocyte markers (CD45) compared with their isotype controls.

The ASCs were isolated and cultivated for approximately 48 h. After this period, most of the cells attached to the culture plate displayed a spindle‐like phenotype. ASCs cells at the second passage were used for flow cytometry analysis to verify their purity. Immune‐phenotyping assays showed that 98.9% of cells expressed CD90 marker, and 94.5% of them expressed CD105 marker. The majority of cells were negative for CD34 (haematopoietic) and CD45 (pan‐leukocyte) markers (Figure 6). These results confirmed that isolated ASCs possessed mesenchymal features.^32^, ^33^

### 3.5 Differentiation of ASCs into keratinocytes

**FIGURE 7:**
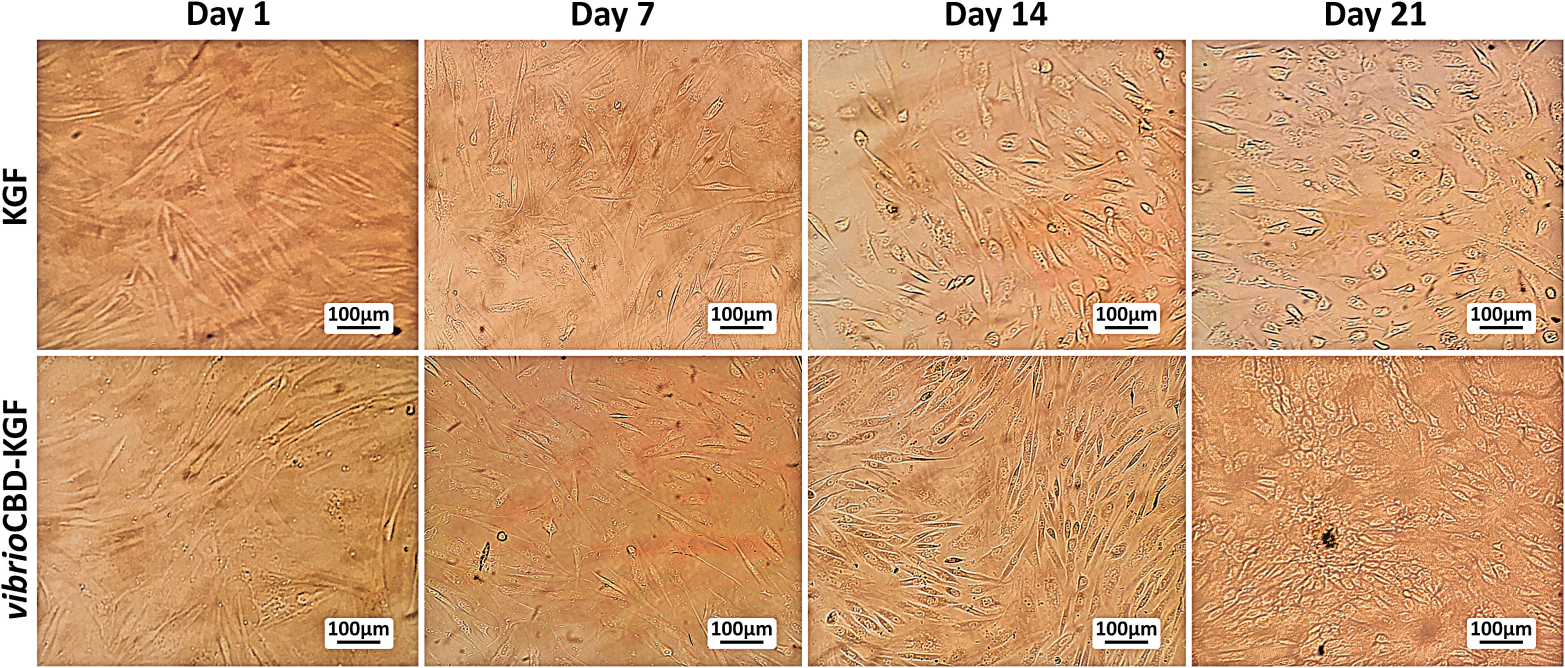
Analysis of morphological appearance of ASCs during induction for differentiation into keratinocytes. ASCs were cultured in differentiation media containing KSFM media plus KGF or *vibrio*CBD‐KGF (20 ng/mL) for 21 days. ASCs on day 1 showed spindle‐shaped morphology. ASCs after 7 days, 14 days, and 21 days in differentiation media displayed morphological changes. (ASCs cultivated with *vibrio*CBD‐KGF showed polygonal‐shaped at day 21 of the differentiation process). Scale bar: 100 μm

ASC cells were cultured in differentiation media containing KSFM supplemented with KGF or *vibrio*CBD‐KGF to differentiate into keratinocytes. Morphological changes in ASC cells were evaluated during the differentiation process (Figure 7). Microscopy analysis showed that ASCs cells maintained their spindle‐like shape when cultured in proliferation media. However, the morphology of ASCs cells cultured in differentiation media containing KGF or *vibrio*CBD‐KGF gradually changed after 7 days. The morphology change was more evident in cells treated with the highest concentration (20 ng/ml) of *vibrio*CBD‐KGF, with some cell populations showing polygonal appearance at the end of the differentiation process (day 21). In contrast, ASC cells cultivated with KGF (all concentrations) or *vibrio*CBD‐KGF at concentrations of 2 and 10 ng/ml showed minor morphological alternations, and their phenotypes were not turned into polygonal shapes (Data not shown).

### 3.6 Analysis of differentiation markers by real‐time PCR

**FIGURE 8:**
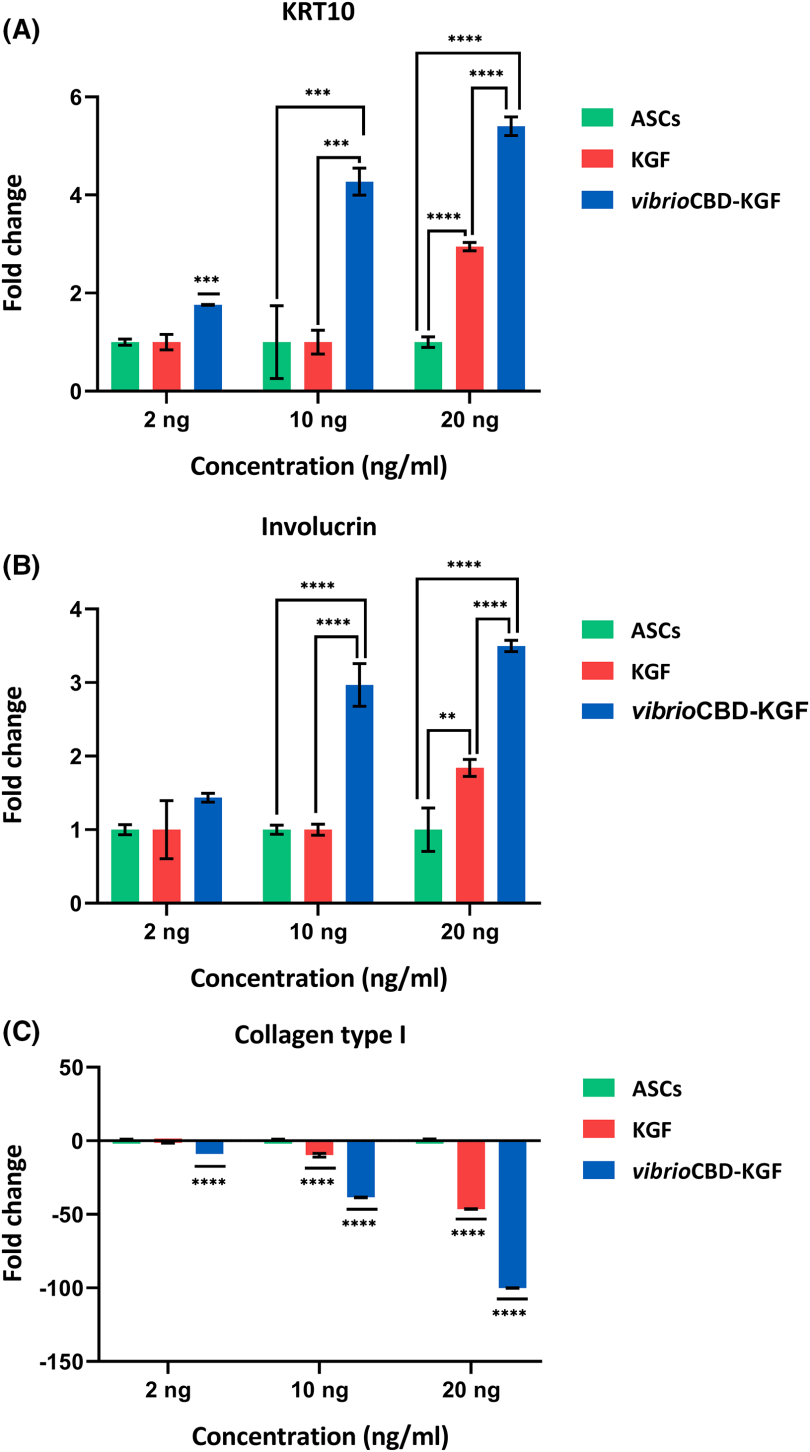
Evaluation of ASCs differentiation into keratinocytes by real‐time PCR. Relative (fold change) gene expression of (A) KRT10 and (B) Involucrinin in ASCs after 21 days of treatment with various concentrations of KGF or *vibrio*CBD‐KGF vs. untreated ASCs. (C) Collagen‐I expression profile was quantified to seek relative fold reduction in stem cell markers during the differentiation process. Down‐regulation of Collagen‐I in ASCs treated with *vibrio*CBD‐KGF was significantly different from untreated control and KGF‐treated ASCs at all tested concentrations. GAPDH was used as an internal control gene. The data shown are mean ± SD (*n* = 3). *p < 0.05, **p < 0.01, ***p < 0.001, ****p < 0.0001 compared to the control group.

The real‐time PCR was used to assess KGF and *vibrio*CBD‐KGF potential in inducing the differentiation of ASCs into keratinocytes. For this purpose, the mRNA expression profiles of two specific keratinocyte markers (KRT10 and Involucrin) were investigated. As shown in Figure 8A,B, a significant enhancement in the expression of KRT10 and Involucrin was only observed at the highest concentration of KGF (20 ng /ml). On the other hand, treatment of ASC cells with *vibrio*CBD‐KGF at all concentrations significantly increased the expression of both the keratinocyte markers. The only exception was a non‐significant increase in the expression of Involucrin at 2 ng/ml of *vibrio*CBD‐KGF. Expression of both keratinocyte markers was increased from the lowest to the highest concentration of *vibrio*CBD‐KGF and was significantly higher than KGF‐treated cells at the same concentration. Interestingly, cells underwent apoptosis when the growth factors were used at a higher concentration of 40 ng/ml (data not shown).

Changes in the expression of Collagen‐I and Vimentin as the marker of undifferentiated MSCs were also evaluated to identify the initiation of the differentiation process. The data demonstrated that Collagen‐I was significantly down‐regulated in both *vibrio*CBD‐KGF and KGF‐treated ASC cells. Nevertheless, the reduction in expression of Collagen‐I was more evident in ASC cells stimulated with *vibrio*CBD‐KGF at the same concentration (Figure 8C). On the other hand, downregulation in the expression of Vimentin was not detected in ASC cells treated with all concentrations of KGF or *vibrio*CBD‐KGF (Figure S2).

### 3.7 Analysis of differentiation markers by immunofluorescence

**FIGURE 9:**
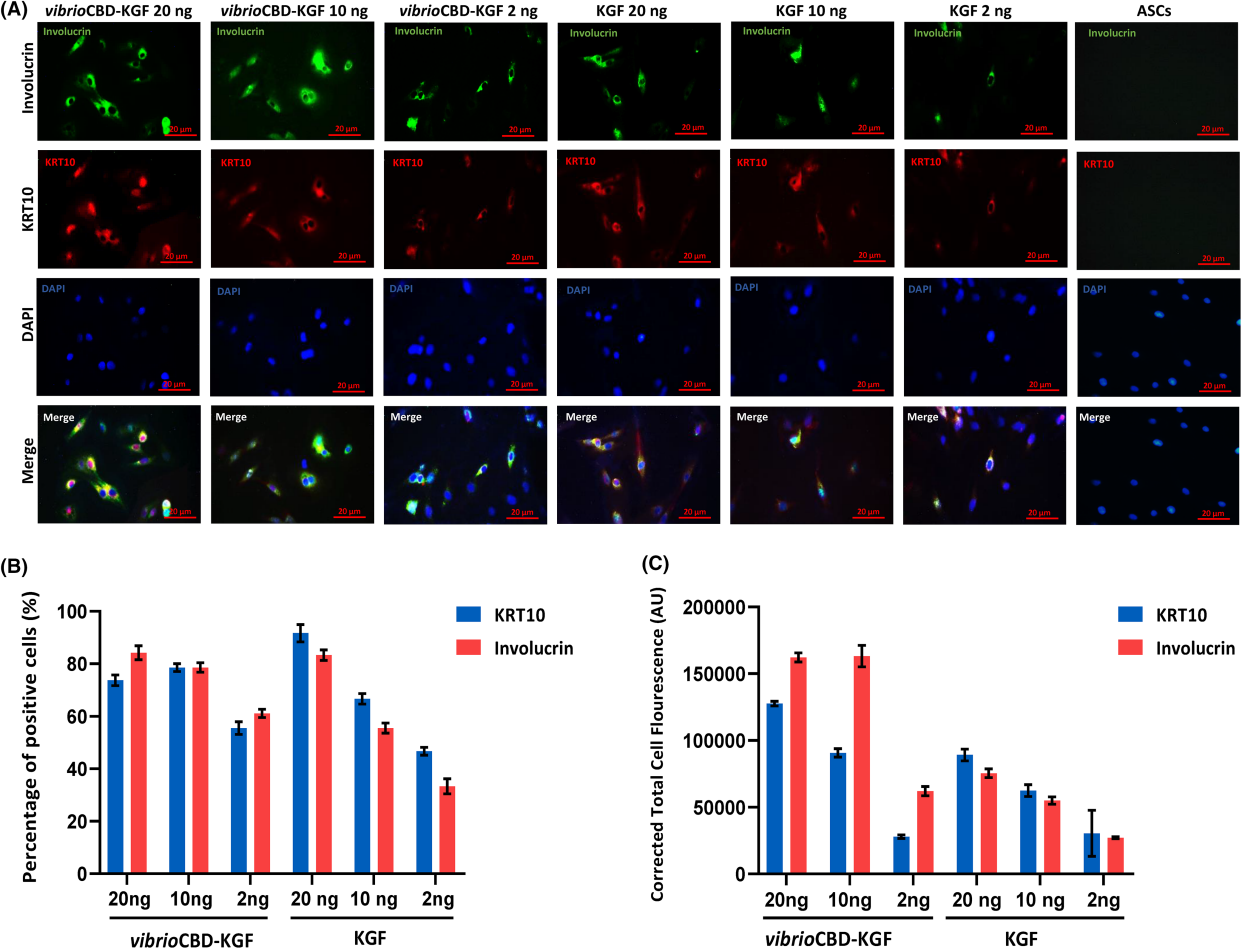
(A) Immunofluorescence staining to evaluate ASC cells differentiation into keratinocytes. ASCs cultured in KSFM media supplemented with different concentrations of *vibrio*CBD‐KGF or KGF (2, 10, and 20 ng/ml) for 21 days. Cells were fixed, and immunofluorescence images were obtained by KRT10 (red) and Involucrin (green) staining. DAPI (blue) was used to visualize nuclei. ASCs cultured in proliferation media were used as the negative control. Scale bar: 20 μm. (B) The percentages of positive cells expressing KRT10 and Involucrin and (C) fluorescence intensity (arbitrary units) were analysed by Image J software. Cell fluorescence was calculated as Corrected Total Cell Fluorescence (CTCF). CTFC = Integrated Density – (Area of selected cell X Mean fluorescence of background readings)

To further investigate the differentiation of ASCs to keratinocytes, immunofluorescence staining was performed to localize KRT10 and Involucrin proteins as markers of keratinocytes. ASC cells cultured for 21 days in differentiation media containing KGF or *vibrio*CBD‐KGF were subjected to an immunofluorescence experiment. Expression of both markers was detectable in the cytoplasm of ASC cells treated with *vibrio*CBD‐KGF at 2 ng/ml with increasing positive staining at the higher concentrations. On the other hand, ASC cells treated with 2 ng/ml of KGF presented weak, if any, staining for both markers. The positive staining was increased in cells treated with higher concentrations of KGF (Figure 9).

## 4 DISCUSSION

Stem cell therapy has emerged as an effective way for the treatment of deep and difficult‐to‐heal wounds. ASCs have gained much attention due to some advantages such as abundance, easy accessibility, high cell yields, and simple isolation procedures, making them an appropriate stem cell source for therapeutic implications.^34^, ^35^ ASCs can differentiate into keratinocytes if treated with various GFs and/or cultured in different media conditions.^36^, ^37^, ^38^, ^39^ Here, we showed that the addition of KGF to the culture media stimulated the differentiation of ASCs into keratinocytes. Furthermore, the fusion of KGF with collagen‐binding domain derived from *Vibrio mimicus* metalloprotease enhanced the differentiation‐stimulating capacity of KGF.

Several studies applied the fusion of a collagen‐binding moiety and a growth factor (CBD‐growth factor) to improve the delivery and biological activity of the GFs. They reported that collagen‐binding ability and biological activity of a CBD‐growth factor are affected by both CBD and growth factor structure.^40^ To date, several collagen‐binding moieties, derived from different sources, have been used for fusing to GFs, including von‐Willebrand factor (vWF),^41^, ^42^ bacterial collagenase,^43^, ^44^ human collagenase,^42^, ^45^ and fibronectin.^46^, ^47^ The choice of the appropriate CBD depends on physicochemical properties of the CBD and growth factor. Fusion with large CBDs such as CBD from *Clostridium histolyticum* collagenase (with a molecular weight of 24 kDa) might decrease the biological activity of the growth factor, probably because of the steric hindrance between bulky CBD and the growth factor that hampers the formation of native three‐dimensional structure of the growth factor.^43^ Besides, glycosylation is required for optimum collagen‐binding of the CBD from Fibronectin.^48^ As a result, Fibronectin CBD is not recommended to produce the fusion protein in *E. coli*.^40^, ^49^ Some studies indicated that the fusion of CBD from *Vibrio* to Epidermal growth factor improved collagen‐binding affinity of the growth factor while leaving the biological activity unaffected.^50^, ^51^ Moreover, in our previous in silico study, 3D structures of different KGF fusions containing CBDs from various sources were predicted, and their affinity binding to collagen and KGFR were estimated. The results showed that CBDs with medium sizes, such as CBD from *Vibrio mimicus*, are most appropriate for fusion to KGF, as large CBDs interfere with receptor binding, and small CBDs result in weak collagen binding.^52^ Accordingly, we decided to use the CBD domain of *Vibrio mimicus* metalloprotease to make *vibrio*CBD‐KGF. The CBD of *Vibrio*, which comprises 33 amino acids, including two essential FAXWXXT motifs,^25^ was placed at the N‐terminal of KGF to increase the collagen‐binding affinity of the molecule.

We produced KGF and *vibrio*CBD‐KGF at 25 and 30°C, respectively, to increase the solubility of proteins. Heparin‐sepharose chromatography followed by SP cation exchange chromatography was applied for purification of KGF, and highly pure protein was obtained with a single band in SDS‐PAGE analysis. On the other hand, we observed that *vibrio*CBD‐KGF lost its affinity for Heparin‐sepharose, probably due to the change in the 3D structure of KGF. We purified *vibrio*CBD‐KGF by hydrophobic interaction chromatography (HIC) that separates molecules based on their hydrophobicity.^53^ Surprisingly, *vibrio*CBD‐KGF attached to the HIC column at a low concentration of NaCl, a weak chaotropic salt,^54^ indicating strong hydrophobicity of the fusion. Most proteins of *E. coli* did not bind to the HIC resin at this condition, allowing us to highly purify the fusion protein in a single chromatography step. *vibrio*CBD‐KGF presented enhanced binding affinity to the collagen‐coated microplate compared to KGF, as shown by collagen‐binding assays. Moreover, *vibrio*CBD‐KGF and KGF displayed comparable biological activities in proliferating HEK‐293 and MCF‐7 cells. It implies that fusion of KGF with CBD of *Vibrio* had no apparent adverse effect on binding of KGF to its receptor. Impairment of heparin‐binding ability of *vibrio*CBD‐KGF while maintaining its receptor binding may be attributed to different positions of the heparin‐binding site (residues113‐133) and receptor‐binding site (residues 37–56) within KGF molecule.^55^, ^56^

To investigate if KGF or *vibrio*CBD‐KGF stimulates differentiation of ASCs into keratinocytes, expression of the specific early and late keratinocyte differentiation markers, i.e., KRT10 and Involucrin, respectively, were analysed by real‐time PCR and immunofluorescence. During re‐epithelialization, the expression of KRT10 in suprabasal layers rises at the beginning of epidermal differentiation. Involucrin, a terminal differentiation keratinocyte marker, is expressed at the epidermis' granular and upper spinous layers.^57^, ^58^ Real‐time PCR analysis of the keratinocyte markers indicated that *vibrio*CBD‐KGF accelerated keratinocyte differentiation at the three applied concentrations of 2, 10, and 20 ng/ml. On the other hand, KGF could only induce differentiation at 20 ng/ml. Of note, we observed that applying higher concentrations of KGF resulted in apoptosis within several days. This observation is in line with other studies that found signalling pathways that resulted in apoptosis are activated at higher concentrations of free GFs.^59^, ^60^ Internalization of activated GF‐receptors and their accumulation in the early endosome is the leading cause of induction of apoptosis.^61^

Besides, we assessed changes in the expression level of the Collagen‐I and Vimentin that were predominantly expressed by MSCs.^62^ It was demonstrated that down‐regulation of MSCs markers could be considered a primary indicator of stem cell commitment.^63^ Our results indicated that Collagen‐I expression was dramatically decreased in cells that undergo the differentiation process, and the level of down‐regulation was correlated with the increase in the expression of keratinocyte markers. However, downregulation of Vimentin expression was not observed in ASC cells treated with KGF or *vibrio*CBD‐KGF across the tested concentrations (Figure S2). This result could be attributed to in‐vitro culture conditions. While Vimentin is normally the specific marker of mesenchymal cells, and is not usually present in normal epithelial cells, its expression was identified in epithelial cells under in‐vitro culture or in tumour cells of epithelial origin.^64^ It has been found that primary keratinocytes were grown on the plate in serum‐free media with low concentrations of Ca^+2^ co‐express Vimentin and Keratins.^65^ A study on corneal epithelial cells identified that these cells express Keratins and Vimentin until they reach high confluency and form a 4–5 layered epithelium.^66^ Moreover, another research showed that peripheral cells of cultured human keratinocytes expressed Vimentin and assumed that it was required for colony growth and keratinocyte migration.^67^

Furthermore, an increase in expression of KRT10 and Involucrin was confirmed by immunofluorescence staining of cells, i.e., ASCs treated with *vibrio*CBD‐KGF showed stronger signals for both KRT10 and Involucrin markers. Morphological changes in ASC cells during differentiation were also evaluated, and polygonal appearance that is the characteristic of keratinocytes was observed in ASCs treated with *vibrio*CBD‐KGF, especially at 20 ng/ml.

The higher potency of *vibrio*CBD‐KGF in inducing ASCs differentiation into keratinocytes may be attributed to immobilizing of *vibrio*CBD‐KGF on collagen. It has been demonstrated that GFs' immobilization leads to enhanced cell signalling and prolonged biological activity of the GFs. Immobilization of GF prevents its internalization upon binding to the receptor, resulting in continued cell signalling and enhanced biological activities compared to soluble GFs.^68^, ^69^, ^70^

Since the high‐affinity receptor for KGF, FGFR2‐IIIb/KGFR, is predominantly expressed by cells of epithelial origin, we wonder if KGF receptors are present on ASCs. Several studies showed a combination of KGF and some stimulating factors induced the differentiation of MSCs towards keratinocyte lineage.^71^, ^72^ Moreover, it was reported that KGF could stimulate proliferation in preadipocyte and endothelial cells despite the absence of direct evidence of expression of KGFR in these cells.^73^, ^74^ Even though a low level of KGFR was shown to be present in the ASCs, a recent study discovered that KGF‐dependent pathway activation in ASCs was mainly attributed to a potential alternative receptor for KGF presented in ASCs.^75^

The present study is valuable in at least two respects. First, we induced the ASCs differentiation into keratinocytes with KGF or *vibrio*CBD‐KGF in the absence of other stimulating factors. Second, we showed that lower amounts of *vibrio*CBD‐KGF, compared to KGF, are required to stimulate ASCs differentiation into keratinocytes, decreasing the cost of highly expensive KGF.

These findings have important implications for skin regeneration and developing suitable skin substitution based on collagen scaffold and GFs. However, further works need to be done to enhance the potency and stability of KGF fusion proteins to decrease differentiation time.

## AUTHOR CONTRIBUTIONS

**Zahra Amidzadeh:** Investigation (lead); writing – original draft (lead). **Setayesh Yasami‐Khiabani:** Investigation (supporting). **Hamzeh Rahimi:** Investigation (supporting). **Shahin Bonakdar:** Investigation (supporting). **Davoud Shams:** Investigation (supporting). **Mahdi Habibi‐Anbouhi:** Formal analysis (supporting); investigation (supporting). **majid golkar:** Supervision (equal); writing – review and editing (lead). **Mohammad Ali Shokrgozar:** Supervision (equal).

## CONFLICT OF INTEREST

The authors declare that there are no commercial or financial conflicts of interest.

## Supporting information


**Figure S1**.

**Figure S2**.

## Data Availability

The data that support the findings of this study are available from the corresponding author upon reasonable request.
