# Supplementary figures and images for: Enhancement of keratinocyte growth factor potential in inducing adipose‐derived stem cells differentiation into keratinocytes by collagen‐targeting
Source: J Cell Mol Med. 2022 Nov 21;26(23):5929–42. doi: 10.1111/jcmm.17619 (PMC9716227; doi:10.1111/jcmm.17619)

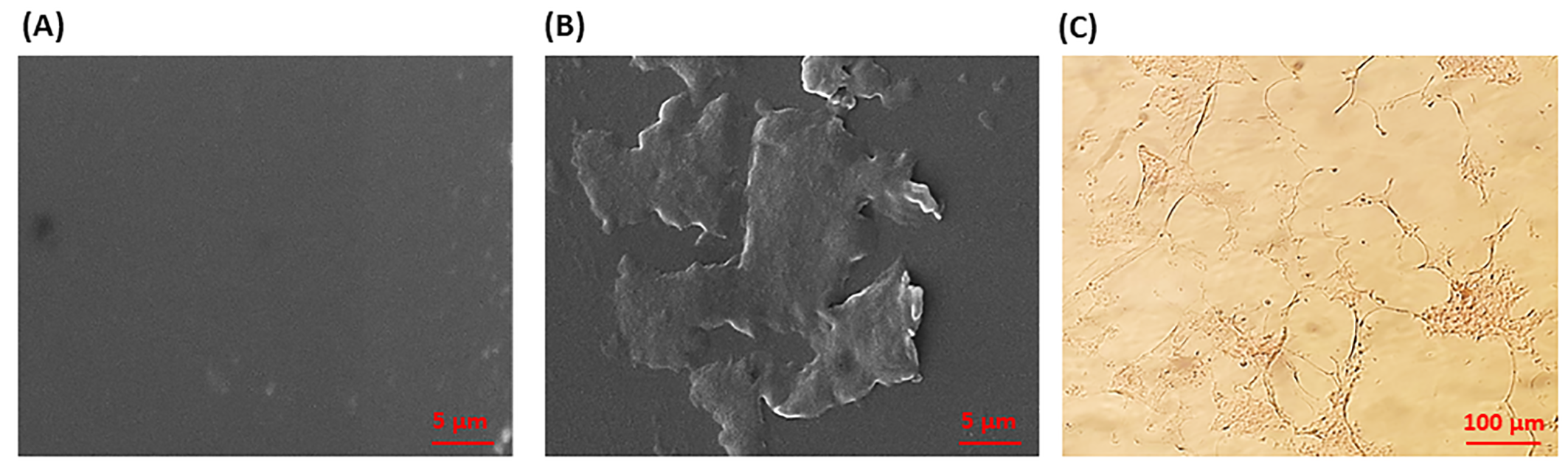

Supplement: Supplementary file 1 — Figure S1. [file JCMM-26-5929-s001.tif]

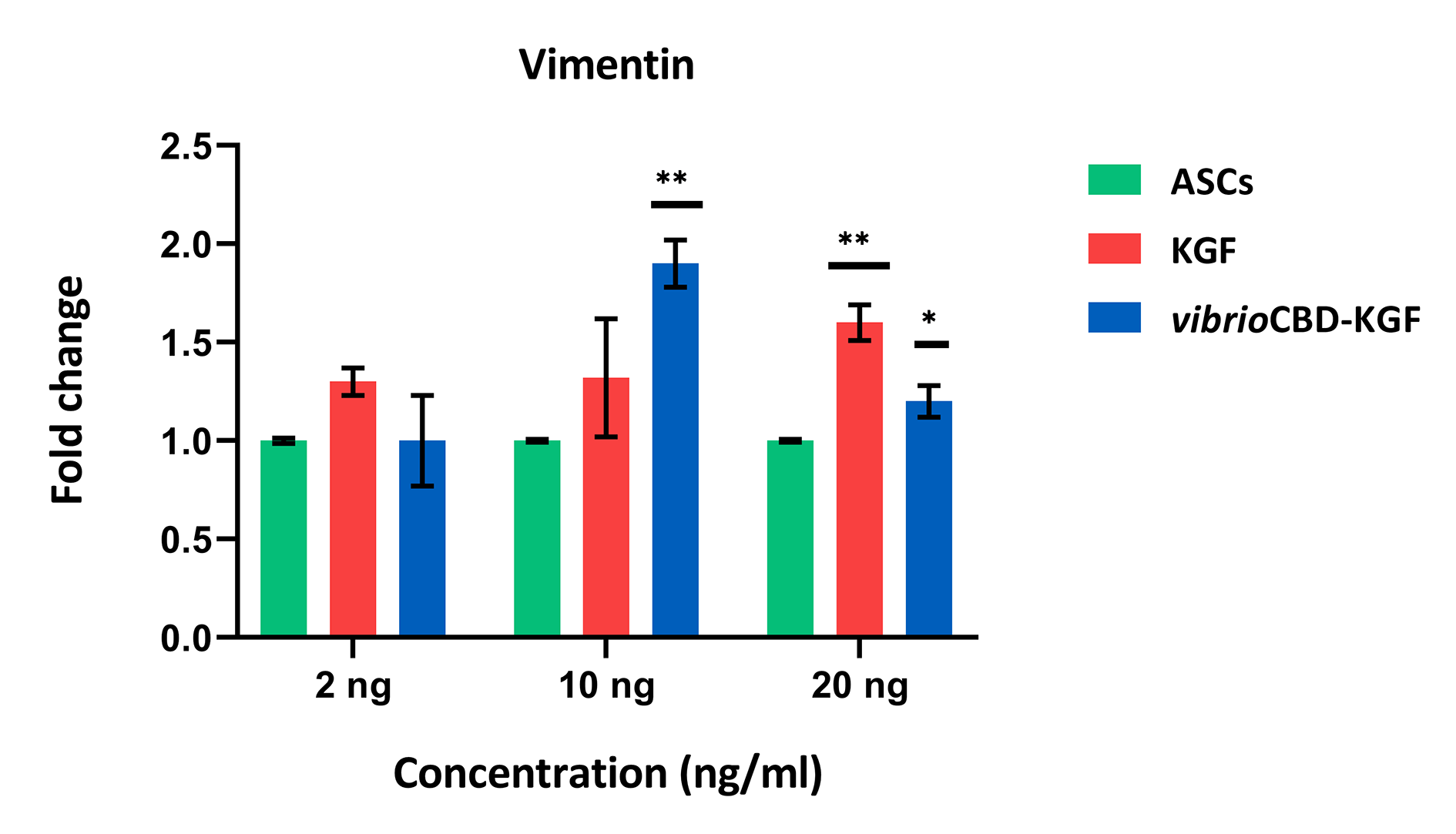

Supplement: Supplementary file 2 — Figure S2. [file JCMM-26-5929-s002.tif]
